# Intracranial Aneurysms in Patients With Coarctation of the Aorta: To Screen or Not to Screen?∗

**DOI:** 10.1016/j.jacadv.2023.100430

**Published:** 2023-07-28

**Authors:** Sara A. Thorne, Beatriz Fernández-Campos

**Affiliations:** Division of Cardiology, University of Toronto, University Health Network and Mount Sinai Health System, Toronto, Ontario, Canada

**Keywords:** coarctation of the aorta, intracranial aneurysm, screening

**Figure:**
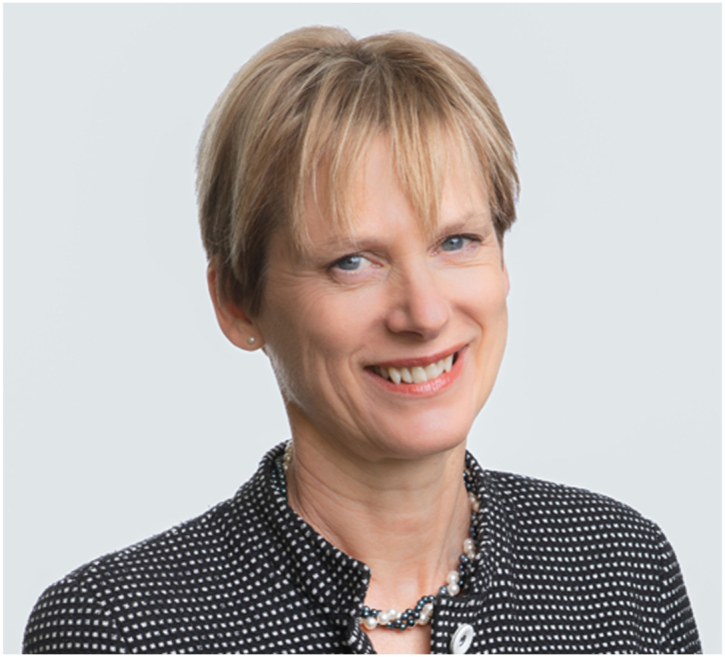

In this issue of *JACC: Advances*, Buckley et al^1^ examine the link between intracranial aneurysms (IAs) and coarctation of the aorta (CoA), and consider the implications for asymptomatic screening. CoA is one of the commonest forms of congenital heart disease, with an estimated prevalence around 4:10,000 live births.^2^ Surgical repair of CoA was first described in 1944,^3^ significantly improving the natural history of the disease. Nonetheless, historical studies reported reduced survival rates even after surgical repair, with a mean age at death of 38 years.^4^ Age at the time of surgical repair emerged as the most important predictor for the development of future hypertension and long-term survival. It is not surprising that with the development and implementation of new diagnostic modalities, and advances in surgical, interventional, and pharmacological strategies, the life expectancy of patients with CoA has been transformed, with an actuarial survival rate of 89% at 60 years.^5^

The association between CoA and IA was first described in 1871 and was followed by several case reports^6^ until 2003, when the first prospective study investigating the relationship between IA and CoA reported an IA incidence of 10% at a mean age of 41.6 years. The study cohort involved 100 patients who may not be representative of contemporary populations: mean age at CoA diagnosis was 17.3 ± 16.3 years and 63% were hypertensive at the time of the study. Subsequent studies have investigated IA in this population and have found conflicting results, with some earlier studies reporting a prevalence of 10%^7^^,^^8^ and other contemporary cohorts reporting a 0% prevalence.^9^ Further evidence suggesting a link between CoA and IA was investigated in a population-based study of patients diagnosed with stroke. It found that stroke patients with CoA had higher rates of subarachnoid hemorrhage when compared to those without coarctation (11.8% vs 4.8%, *P* = 0.039), and that among patients with subarachnoid hemorrhage, the prevalence of unruptured IAs was higher in patients with CoA compared with those without (23% vs 2.5%, *P* = 0.002).^10^ Hypertension has been recognized as a risk factor for the development and rupture of IA in the general population^11^; however, the impact of the age at hypertension diagnosis or antihypertensive medical treatment has been inconsistently studied in these cohorts.

In this issue of *JACC: Advances*, Buckley et al^1^ present the findings of their meta-analysis investigating the prevalence and risk factors for IA in patients with CoA. The authors included five studies published over a 17-year span. They included 442 patients, of whom 27 were diagnosed with an IA, estimating a pooled prevalence of 3.8% (95% CI: 0.1%-12.3%), similar to the 3.2% prevalence in the general population.^11^ Buckley et al^1^ also highlight the role of modifiable risk factors in the development of IAs in patients with CoA. Among the available risk factors, the presence of systemic arterial hypertension was the only univariate predictor of IA, with an odds ratio of 3.1 (95% CI: 1.1-8.2; *P* = 0.03).

The authors emphasize the down trending incidence of IAs between earlier and modern studies, with the latter reporting a lower IA rate. We agree with Buckley et al in that CoA is frequently diagnosed even before birth, and interventions are performed earlier in life; moreover, the increasing numbers of specialized adult congenital heart disease centers, the known relationship of CoA with the development of systemic arterial hypertension, and the deleterious effects of the latter, have prompted better surveillance and treatment strategies in this population. An earlier age at CoA diagnosis and treatment could also reflect lower coarctation-years and hypertensive-years exposure, likely reducing the risk of the development of future IAs.

The American Heart Association 2015 stroke guidelines suggest that an aneurysm measuring <7 mm is associated with a very low risk of rupture.^11^ A more recent study that followed 1,960 aneurysms for 7,388 aneurysm years found that aneurysms ≥5 mm were associated with increased risk of rupture when compared to aneurysms of 2 to 4 mm.^12^ Surgical or percutaneous treatment strategies have high morbidity and mortality risks and are only recommended in patients with high-risk features.^11^ Since this meta-analysis by Buckley et al^1^ demonstrates a very low rate of detection of clinically significant aneurysms, with only 5 (1.1%) measuring ≥5 mm, it brings into question the true benefit of asymptomatic screening in this population.

In this vein, a cost analysis study suggested that screening for IA in patients with repaired CoA was cost-effective at 10, 20, and 30 years of age in a simulated cohort.^13^ However, the authors used an estimated IA prevalence of 10%, which likely reflects an overestimate in a contemporary population of patients with repaired CoA. Furthermore, previous studies have failed to detect IAs in younger patients with CoA (mean age at brain imaging: 16 years),^14^ which also conflicts with this cost analysis study. Current guidelines are also discrepant with regard to IA screening: the American Heart Association suggests that screening of IA in patients with CoA may be reasonable,^15^ whereas the European Society of Cardiology suggests that routine screening is not recommended.^16^

To answer our question, “To screen or not to screen?”, we conclude that the prevalence of IAs in patients with CoA who have received the current standard of care is similar to the prevalence of IAs in the general population (Figure 1). A universal screening strategy is therefore likely to detect mostly small, nonclinically relevant IAs, causing anxiety and being cost ineffective, overutilizing already stretched medical systems. Furthermore, the rate of IA progression, and hence an ongoing surveillance strategy in this scenario has not been well described.

**Figure 1:**
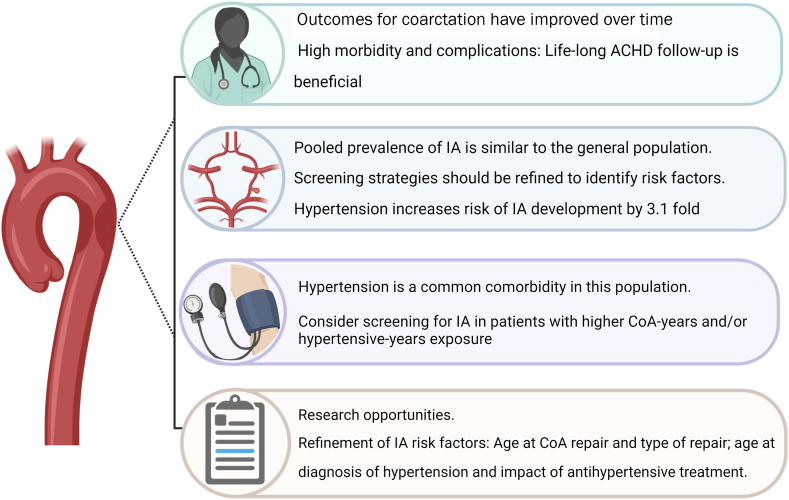
**Intracranial Aneurysms in Patients With Coarctation of the Aorta** Created with BioRender.com. ACHD = adult congenital heart disease; CoA = coarctation of the aorta; IA = intracranial aneurysm.

As Buckley et al^1^ conclude, indiscriminate IA screening in this population is not supported by the data. Perhaps a more efficient way to address screening for IA in patients with CoA is to consider the population truly at risk, that is, those with a high hypertensive-years exposure. Patients with older age at CoA diagnosis or CoA treatment and those with long-standing uncontrolled or untreated systemic arterial hypertension could potentially benefit from this strategy. Further studies are needed to define the impact of screening the CoA population at risk of IA development, growth, and rupture.

## Funding support and author disclosures

The authors have reported that they have no relationships relevant to the contents of this paper to disclose.
